# Longitudinal evaluation of the Ophthalmology residents in Brazil: an observational prospective study

**DOI:** 10.1590/1516-3180.2022.0092.R1.01072022

**Published:** 2022-10-03

**Authors:** Josie Naomi Iyeyasu, Dario Cecilio-Fernandes, Keila Monteiro de Carvalho

**Affiliations:** I MD. Ophthalmologist and Assistant Doctor, Low Vision and Strabismus Sector, Hospital de Clínicas, Faculdade de Ciências Médicas da Universidade Estadual de Campinas (HC/FCM/UNICAMP), Campinas (SP), Brazil.; II Psy, MSc, PhD. Psycologist and Researcher, Department of Psychology and Psychiatry, Faculdade de Ciências Médicas da Universidade Estadual de Campinas (FCM/UNICAMP), Campinas (SP), Brazil.; III MD. Ophthalomologist and Full Professor, Faculty of Medical Sciences, Universidade Estadual de Campinas (UNICAMP); and Chief Department of Ophthalmo-Otorrynolaringology, Faculdade de Ciências Médicas da Universidade Estadual de Campinas (FCM/UNICAMP), Campinas (SP), Brazil.; Universidade Estadual de Campinas, Faculdade de Ciências Médicas, Chief Department of Ophthalmo-Otorrynolaringology, Campinas, SP, Brazil

**Keywords:** Ophthalmology, Education, medical, Internship and residency, Knowledge assessment, Longitudinal evaluation, Online test, Residency, Medical residency

## Abstract

**BACKGROUND:**

The longitudinal evaluation of students seems to be a better way to assess their knowledge compared with that of the traditional methods of evaluation, such as modular and final tests. Currently, progress testing is the most consolidated type of longitudinal testing method. However, despite being well consolidated as an assessment tool in medical education, the use of this type of test in residency programs is scarce.

**OBJECTIVES:**

This study aimed to investigate residents’ knowledge growth regarding residency training and to describe the implementation of a longitudinal evaluation test in ophthalmological residency training across several medical schools in Brazil. Finally, the study aimed to check whether performance in the tests can be used as a predictor of the results of the specialist title test.

**DESIGN AND SETTING:**

This was a prospective observational study. This study was conducted using an online platform.

**METHODS:**

Online tests were developed following the same pattern as the Brazilian Ophthalmology Council specialist tests. All the residents performed the test simultaneously. The tests were conducted once a year at the end of the school year.

**RESULTS:**

A progress test was conducted across 13 services with 259 residents. Our results demonstrated that resident scores improved over the years (P < 0.0001) and had a moderate correlation with the Brazilian Opthalmology Council specialist test (P = 0.0156).

**CONCLUSION:**

The progress test can be considered a valuable tool to assess knowledge, meaning their knowledge increased over residency training. In addition, it can be used as a predictor of the result in the specialist title test.

## INTRODUCTION

Knowledge assessment plays an important role in medical education since professional expertise development appears to be strongly connected to knowledge.^1^ Research has shown that assessment may be used in different ways. For example, studies have demonstrated that assessment drives and stimulates learning,^2,3^ provides educational efficacy information to institutions and teachers, and protects patients.^1^

The definitions of “to test” in the dictionary are as follows: to discover the worth of something by trial, to obtain more information about the object of assessment, and to improve the quality of something by trial.^4^ Thus, assessment in the broader sense involves testing, measuring, collecting, combining information, and providing feedback.^4^

In many medical residency programs, modular, intermediate, or final tests have been used to measure the knowledge level of trainees.^5,6^ However, these types of tests are associated with the promotion of short-term memorization.^5^ In addition, residents' performance may not correspond to the real knowledge level since it is merely a one-point measurement, not allowing any extrapolation to the maintained knowledge level over time.^7^ To benefit students' long-term retention, longitudinal testing in the form of the progress test, the most known and established kind of longitudinal test, has been suggested.^2,8^

Progress testing aims to measure students' knowledge at the end level and allows the measurement of knowledge growth.^8,9^ In addition, progress testing forces students to study over time, encouraging more profound and deep learning^10^ since it is impossible for students to cram before the test. Alternatively, students must acquire information continuously in such a way that it is available when required.^11^ Progress tests allow for individual learning pathways, which may provide clues for future performance. Finally, progress testing can be organized at a national level^7^ and can be used to compare the results of candidates from different countries.^5^

Progress tests have been used in different ways, such as for providing feedback to students,^12,13^ understanding knowledge growth on questions requiring lower and higher order of cognitive processing,^12,13^ comparing national^14^ and international curricula,^15^ and the effectiveness of educational strategies.^16,17^ Many medical schools worldwide have already adopted this progress testing as part of their curricula, such as the Netherlands,^18^ Canada,^19^ Germany,^6^ Indonesia, South Africa, the United States,^20^ and Brazil.^21,22^

Despite being a well-established assessment tool in the undergraduate context, progress testing is much less widespread in the postgraduate context, where the best test format remains controversial.^23^ Some authors believe that, at least in theory, longitudinal tests would also be an interesting approach to knowledge assessment in postgraduate medical education.^7^ So far, only a few residency programs have already included the progress test in their curricula, such as in obstetrics and gynecology,^24^ radiology, and in general practice,^10,25^ demonstrating promising results.

The World Reference Institution in ophthalmology residency programs is the International Council of Ophthalmology (ICO). According to the ICO, medical knowledge is one of the general core competencies expected from ophthalmic specialists (besides patient care, practice-based learning and improvement, communication skills, professionalism, and systems-based practice).^26,27^

Progress testing during residency could play an important role in monitoring the competence progress. Besides, it could be useful for the quality control of residency programs in Brazil, by allowing interventions during the course. In addition, the tests can serve as self-learning tools for residents. Finally, it can be useful to predict residents' results in the specialist test of the Brazilian Opthalmology Council.^28^

## OBJECTIVE

This study aimed to investigate residents' knowledge growth during their residency training. This study also describes the implementation of a progress test in ophthalmological residency training across several medical schools in Brazil. Finally, this study aimed to investigate whether there was a correlation between the performance of the progress test and the specialist title test.

## METHODS

This was a prospective observational study carried out through an online platform.

This study was approved by the ethics committee of Universidade Estadual de Campinas on December 17, 2018 (CAAE number:02613718.9.0000.5404).

Participants: The study was conducted in 2019. All participants were ophthalmology residents who agreed to participate voluntarily in the study and signed a consent form.

### Ophthalmology Residency in Brazil

In Brazil, the ophthalmology residency consists of a 3 years program.

The institution that represents the Brazilian Ophthalmology is the Brazilian Ophthalmology Council (Conselho Brasileiro de Oftomologia, CBO).^28^ According to the CBO, the minimum pedagogic program required for the ophthalmology specialization consists of the following content:

1. Basic sciences: 100% in the 1^st^ year and 0% in the 2^nd^ and 3^rd^ year
2. Propaedeutics: 60% in the 1^st^ year, 30% in the 2^nd^, and 10% in the 3^rd^ year
3. Optometry: 50% in the 1^st^ year, 50% in the 2^nd^, and 0% in the 3^rd^ year
4. Surgical techniques: 50% in the 1^st^ year, 50% in the 2^nd^, and 0% in the 3^rd^ year
5. Clinics and surgery: 25% in the 1^st^ year, 50% in the 2^nd^, and 25% in the 3^rd^ year

Besides this mandatory content, there may be complementary activities, such as clinical case discussions, pathological anatomy sections, and scientific article discussions.^28^

### Progress test construction and application

The progress test consisted of 125 multiple-choice questions on clinical and surgical issues in ophthalmology. The blueprint followed the same pattern as the Brazilian Ophthalmology Council specialist test:^28^

- uveitis: 9 questions;
- neuro ophthalmology: 7 questions;
- orbit: 4 questions;
- lacrimal system: 4 questions;
- ocular plastics: 8 questions;
- ocular tumors: 5 questions;
- cornea: 14 questions;
- contact lenses: 4 questions;
- refractive surgery: 2 questions;
- retina: 13 questions;
- cataract: 10 questions;
- glaucoma: 11 questions;
- refraction: 23 questions;
- strabismus: 7 questions;
- low vision: 4 questions.

The Graphic 1 shows the division of the test questions.

**Graphic 1:**
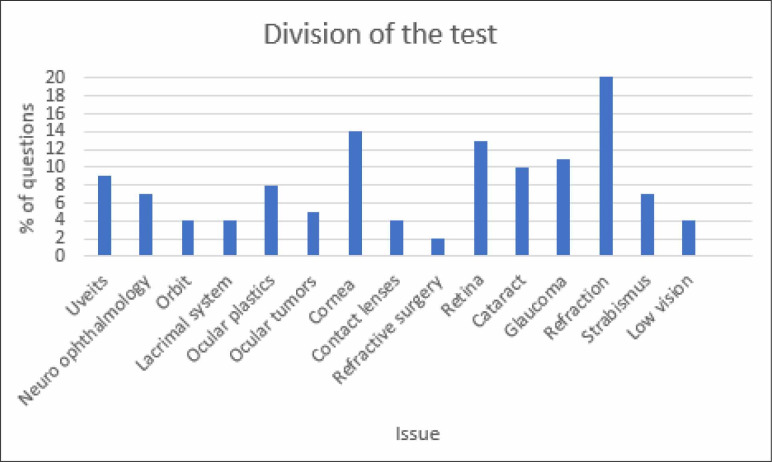
Division of the test questions.

As the tests consisted of 125 multiple-choice questions, for the statistical analysis, a 0.08 value corresponds to 10/125 for each correct answer; thus, it was attributed to a score that could vary from 0 to 10 for each test.

The questions in the tests were taken from the following books: Review Questions in Opthalmology,^29^ Clinical Optics and Refraction,^30^ and Self-tests in Optic and Refraction.^31^ They were chosen according to the issue and level of difficulty (judged by the authors), in a way that there were questions of different issues and levels of difficulty.

As there were residents from many parts of the country, the tests were online, and all the residents from the 1^st^ to the 3^rd^ year of the ophthalmology residency programs performed the tests simultaneously. Therefore, all residents were enrolled in the same test, regardless of whether they were in their 1^st^, 2^nd^, or 3^rd^ year of residency. The tests were conducted once a year at the end of the school year.

Each service organized the implementation of the tests, and the only requirement was that all residents sat on the test simultaneously. Some services used their own informatic lab rooms, while those that did not have one allowed their residents to use their own computers, either at the service or at home, at a predetermined schedule, as long as there was one computer for each resident.

### Site

**Figure 1:**
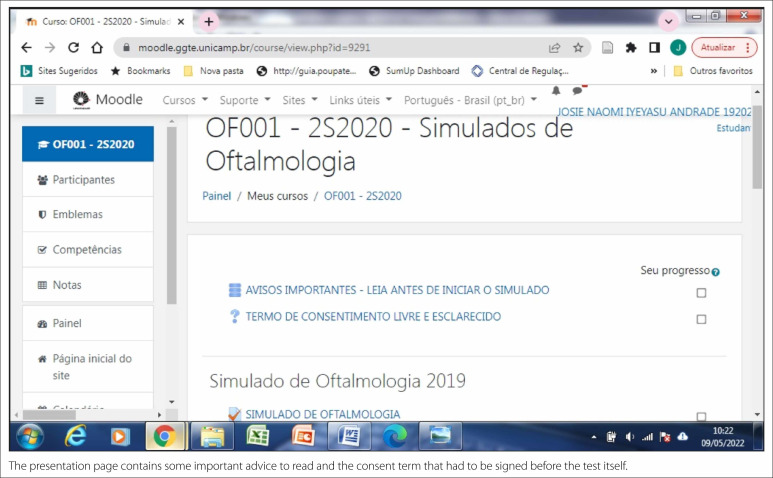
Presentation page.

**Figure 2:**
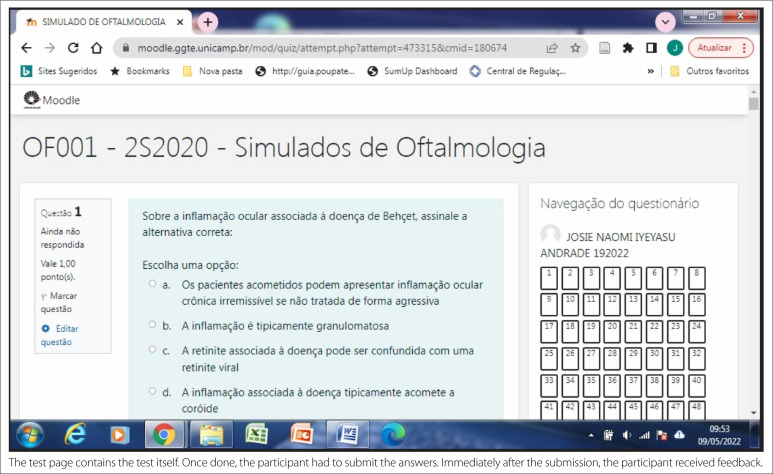
Test page.

**Figure 3:**
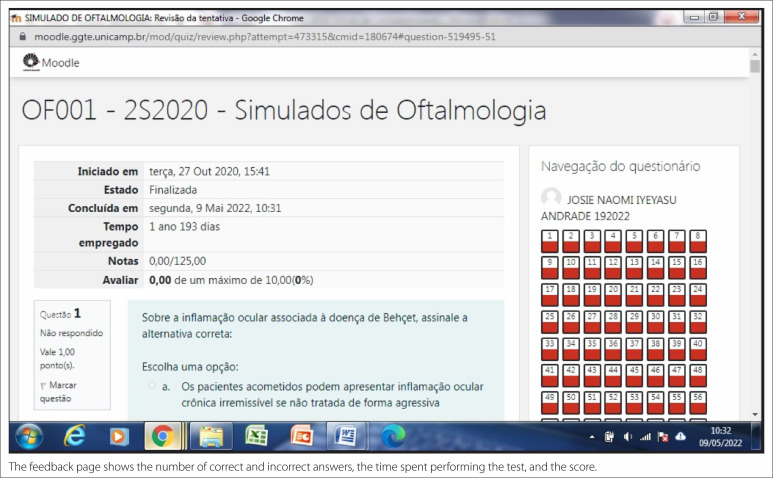
Feedback page.

**Figure 4:**
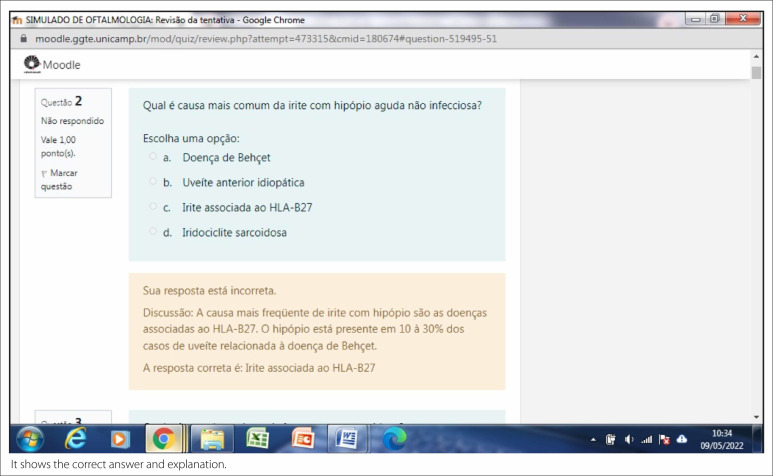
Example of a discussion feedback.

First, participants had to create an account. Once completed, they were able to access the site. Figures 1–4 show a small portion of the site.

The presentation page contains some important advice to read and the consent term that had to be signed before the test itself (Figure 1).

The test page contained the test itself. Once completed, participants had to submit their answers. Immediately after the submission, the participant received feedback (Figure 2).

The feedback page shows the number of correct and incorrect answers, the time spent performing the test, and the score (Figure 3).

Figure 4 shows the correct answers and explanations.

### Data analysis

Frequency tables were used for the descriptive analysis of categorical variables. Positions and dispersion measures were used for numeric variables. The Kruskal–Wallis test was used to compare the differences between years, followed by Dunn's test to identify significant differences.

The Friedman or Wilcoxon test was used to compare students' knowledge growth.

To investigate the relationship between the progress test and CBO scores, the Spearman linear correlation coefficient and Wilcoxon test for related samples were conducted.

A statistical level of 0.05 was considered significant.

Data were analyzed using the Statistical Analysis Software (SAS) System for Windows (Statistical Analysis System), version 9.4. SAS Institute Inc, 2002-2012, Cary, North Carolina, United States.

## RESULTS

Among the many ophthalmology residents all around Brazil invited to join the study, 24 accepted the invitation. A total of 297 residents participated in the progress test. Of these, 100 (33.7%) were from the 1^st^ year, 108 (36.4%) from the 2^nd^ year, and 89 (30.0%) from the 3^rd^ year.

### Descriptive analysis and comparison of the scores for each residency year

The mean score of the 1^st^ year residents was 4.3, that of the 2^nd^ year residents was 5.1, and that of the 3^rd^ year residents was 5.4. Table 1 and Graphic 2 show the descriptive analysis and comparison of scores for each residency year.

**Graphic 2:**
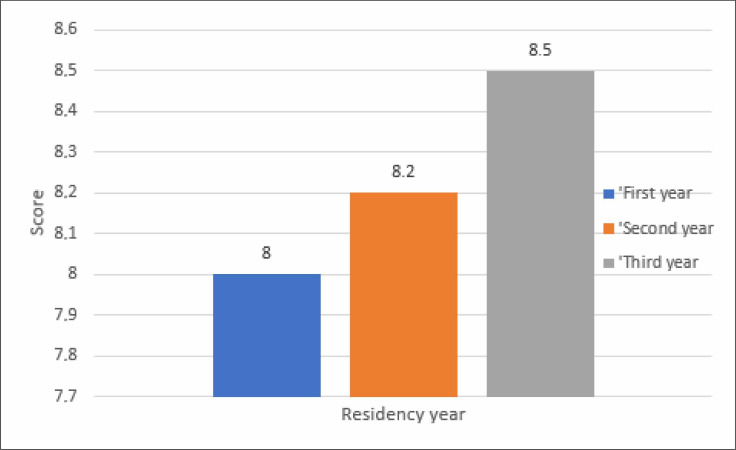
Descriptive analysis of the scores for each residency year.

**Table 1:** Descriptive analysis and comparison of the scores for each residency year

Residency year	n	Mean score	SD	Minimum score	Median	Maximum score
1^st^ year	97	4.3	1.0	2.3	4.2	8.0
2^nd^ year	104	5.1	1.2	2.7	5.0	8.2
3^rd^ year	89	5.4	1.1	3.4	5.4	8.5

n = number of participants; SD = standard deviation.

The Kruskal–Wallis test was used to compare the mean scores across the three years of residency. The P value was < 0.0001, which was considered statistically significant. Therefore, it is possible that there was a difference between the mean scores.

The Wilcoxon test was used for multiple comparisons of the mean scores for each pair of the residency years (1^st^ versus 2^nd^, 1^st^ versus 3^rd^, and 2^nd^ versus 3^rd^) to check the difference between the pairs. There was a significant difference between the 1^st^ and 2^nd^ years and the 1^st^ and 3^rd^ years of residency (P < 0.0001 in both cases). However, the difference between the 2^nd^ and 3^rd^ years of residency was not significant (P = 0.0619). This may be because of the pedagogic program itself since, if we look at it, we can see that almost all the theoretical content was taught in the first two years of residency, with only a small percentage remaining in the 3^rd^ year of residency.

### Relationship between the progress test and Brazilian Ophthalmology Council (CBO) scores (Table 2 and Graphic 3)

**Graphic 3:**
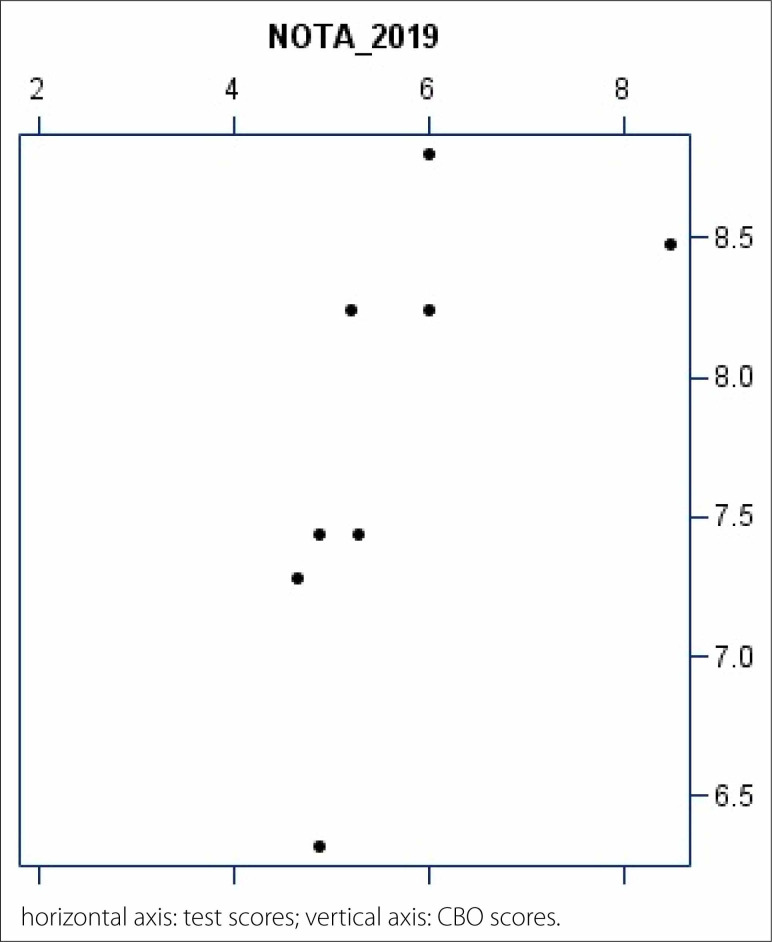
Spearman linear correlation between the progress test and Brazilian Ophthalmology Council (CBO) scores.

**Table 2:** Relationship between the progress test and CBO scores

Variable	n	Mean score	SD	Minimum score	Median	Maximum score
Test score 2019	8	5.7	1.2	4.6	5.2	8.5
CBO score 2019	8	7.8	0.8	6.3	7.8	8.8
dif		2.1	1.0		2.4	

CBO = Brazilian Ophthalmology Council; n = number of participants; SD = standard deviation; dif = difference between the CBO and progress test scores (CBO score–test score). P value = 0.0156 (Wilcoxon test).

For this analysis, we had only eight residents from the 3^rd^ year. Correlation analysis demonstrated an association between the progress test and CBO scores. Spearman correlation (Graphic 3) showed a positive and significant correlation between these two scores (which was 0.61), which means that the higher the score on the progress test, the higher the score on the CBO test.

## DISCUSSION

In this study, we demonstrated that progress tests could be used for ophthalmology residency training. They helped to detect the residents' knowledge growth over time and had a moderate relationship with the CBO test. Our findings are aligned with previous studies in both undergraduate^8,9,32^ and residency training.^10,30–33,34^

For example, in a study by Tomic et al., 4 years of progress testing were evaluated in a medical school in Brazil and positive results were found, with a continuum of cognitive gain during medical training.^32^

Similarly, previous studies with longitudinal tests on the residency program^10,29,30^ found that the progress test was able to detect the difference^33,35^ among residency years. Taken together, the knowledge scores increased over the years. ^10,35^

Concerning the relationship between the progress and CBO tests, our results were partially in concordance with those of previous studies. For example, in an undergraduate context, a study by Hamamoto Filho et al. found a correlation between students' progress testing scores and their performance in a residency selection process in Brazil.^36^

In the residency context, a descriptive study by Al-Mohammed A et al.^37^ compared the residents' performance on the American College of Physicians (ACP) Internal Medicine In-Training Examination (IM-ITE) results and the certification examination of the American Board of Internal Medicine (CABIM) and American Board of Surgery Qualifying Examinations in Qatar, found that the performance on the ITE could accurately predict the performance on both qualifying exams,^31^ which is in concordance with our results.

Therefore, our study is in concordance with previous studies performed by residents. What makes our study exclusive is that besides being performed in a country where there are almost no similar studies, it is, as far as we are concerned, the only one performed with ophthalmology residents.

### For the future

Two more different tests will be developed, and each test will be used at the end of the school year by all the residents from the 1^st^ to the 3^rd^ year of the ophthalmology residency programs.

All the tests will have the same number of questions (125). They will follow the same division of national testing issues; however, the questions will be completely different from one test to another. In other words, all questions will be changed from the 1^st^ year to another. Thus, at the end of the 3 years of residency, each resident performed three different tests.

After the end of the tests, the tests will be revised, and each resident will receive individual performance feedback through an online program developed with personal login and password.

### Limitations of the study

In some services, the residents were allowed to do the test at home because the service did not have informatics labs or an appropriate classroom for them to perform the tests. This can be biased because we cannot guarantee they did not cheat on the test. In addition, as participation in the study was voluntary and the progress test score was not part of the official residency program, some residents did not take it seriously. Finally, our sample size for comparison of the progress and CBO tests was small. However, even with such a small sample size, we found a moderate and significant correlation.

## CONCLUSION

Based on the data obtained, it is possible to see that the scores of the residents improved over the years, which means that their knowledge increased. In other words, there was progress along the residency course.

Residents approved the longitudinal test as a self-learning tool and as a tool for improving residency programs. Therefore, we can say that the implementation of a longitudinal evaluation system in ophthalmological residency schools in Brazil was successful and could be implemented in other medical subspecialties.
